# Bone ingrowth into open architecture PEEK interference screw after ACL reconstruction

**DOI:** 10.1186/s40634-020-00285-z

**Published:** 2020-09-18

**Authors:** Martin Lind, Torsten Nielsen, Ole Gade Sørensen, Bjarne Mygind-Klavsen, Peter Faunø, Stacy Leake-Gardner

**Affiliations:** 1 Department of Orthopedics, Aarhus University Hospital, Palle Juul-Jensens Boulevard 99, 8200 Aarhus N, Denmark; 2 Smith & Nephew Clinical, Scientific & Medical Affairs, Global R&D, 7135 Goodlett Farms Parkway, Cordova, TN 38016 USA

**Keywords:** ACL reconstruction, Graft fixation, Interference screw, Bone ingrowth

## Abstract

**Purpose:**

Open or fenestrated interference screw design that allow bone ingrowth is a concept for improved bone healing to softtissue graft and bone filling in bone tunnels after anterior cruciate ligament reconstruction (ACLR) The aim of the current study was to assess CT scanning evaluated bone ingrowth into an open architecture interference screws in the tibial tunnel of patients undergoing ACL with soft tissue grafts. It was hypothesized that open architecture interference screws would stimulate bone ingrowth into the screw cavities.

**Methods:**

Twelve patients requiring ACLR were included. They underwent arthroscopic ACLR with semitendinosus−/gracilis tendon graft and an open architecture polyetheretherketone (PEEK) interference screw. The patients were scanned with a multi-slice CT scanner two weeks, six and twelve months postoperatively. On CT reconstruction slices bone ingrowth into the screw was measured. Subjective and objective clinical outcome international knee documentation committee score and instrumented knee laxity determination were collected.

**Results:**

At six months no implants demonstrated more than 10% bone ingrowth. At twelve months 42% (5/12) implants had more than 10% bone ingrowth (*p* = 0.009). The average bone filling into the screws was 7.7%. There was no tunnel widening or cyst formation seen in relation to any of the implants. Subjective IKDC score improved significantly from 50.6 baseline to 80.1 at 24 month follow-up. Preoperative side-to-side knee laxity improved from 3.7 (2.1) to 1.4 (1.2) mm at twelve months. There were no serious adverse events in relation to the new open architecture thread PEEK interference screw during or after hamstring ACL reconstruction.

**Conclusion:**

The present study demonstrated that open architecture thread PEEK interference screw can stimulate bone ingrowth into the screws after soft tissue ACL reconstruction with at 12 months with an average bone filling into screws was 7.7%. Knee stability, functional, subjective and objective outcomes were similar to large volume ACL outcome studies.

**Trial registration:**

The study was registered at ClinicalTrials # NCT02382341. 12-09-2014.

**Level of evidence:**

IV.

## Introduction

Advances in Anterior Cruciate Ligament Reconstruction (ACLR) techniques over the last three decades have resulted in 80–100% of patients reporting normal to nearly normal outcome scores following ACLR [16]. Despite these advances, it has been reported that 3–10% of ACL reconstructions fail [14]. A common factor found in revisions is the issue of fixation of the graft to the bone surface in the tibial and femoral tunnels [5, 17, 20]. The long-term success of ACL repair depends on its ability to heal in the bone tunnel [10].

Soft tissue grafts have been proven capable of similar functional and strength outcomes and with less donor site morbidity compared to patellar tendon autografts [25]. But according to recent registry and meta-analysis studies patellar tendon autografts have a significantly lower rate of graft failure and better knee stability than soft tissue graft [8, 9, 21]. The decreased stability of hamstring reconstructions compared to BTB could result from differences in graft properties that adversely affect the ability of the soft tissue graft to heal inside bone tunnels [15, 21].

Fixation of soft tissue ACL grafts in bone tunnels is commonly accomplished by interference screws. Interference screws are made from various materials, including metals, absorbable ceramics and inert polymers. Bio-absorbable screws offer advantages over metallic screws, including decreased graft trauma and better shear strength fixation [3]. However some bioabsorbable materials have been associated with bone resorption due to acidic degradations products [4]. Such bone resorption can cause cyst formation and tunnel widening resulting in increased graft laxity and failure [6]. So far no bioresorbable implant has proven the ability to induce bone formation during implant resorption as initially envisioned at conception for resorbable implants [23].

Another concept for combining improved healing to soft tissue graft with bone filling of the bone tunnels is interference screws with open or fenestrated design that allows bone ingrowth. The concept has been tested in an ovine animal model with resorbable screw material and bone ingrowth into screw perforations were demonstrated [11]. In the clinical setting, bone ingrowth into a soft tissue ACL graft interference screw could result in improved bone to graft healing and easier revision conditions after traumatic graft failure. This is due to improved bone stock and the potential benefit of avoiding screw removal since a low material density interference screw with bone ingrowth may just be drilled through at an optimal anatomical drilling direction.

No human clinical studies have investigated the bone ingrowth potential of open architecture interference screws.

Smith and Nephew has developed the Biosure Healicoil interference screw as a novel approach to interference screws. Made of non-absorbable but radiolucent Polyetheretherketone (PEEK) material, the screw features a fenestrated design providing circumferential contact with the tibial tunnel. The screw’s open profile displaces less surface area than a standard metal or PEEK interference screw while maintaining comparable insertion and fixation strengths. Furthermore, the open design of the Biosure Healicoil interference screw allows bone ingrowth of the bone tunnel without the uncertainty and delay from the resorption and remodeling processes of absorbable and osteoconductive screws.

The primary objective of the current study was to assess via CT scanning bone ingrowth into the Biosure Healicoil interference screw in the tibial tunnel of patients undergoing ACL reconstruction with soft tissue grafts. It was hypothesized that the open architecture screws would result in bone ingrowth into screw voids.

## Materials and methods

This study was a prospective case series. Patients requiring a soft tissue graft ACL reconstruction after ACL injury received a hamstring graft ACLR using the Biosure Healicoil PEEK Interference Screw for tibia fixation and followed for 24 months post-operatively. CT scan imaging evaluated bone ingrowth into the Biosure Healicoil Interference screw at two weeks, six and twelve months. Patient reported outcomes and knee stability was evaluated after six, twelve and 24 months. Men and women between the ages of 18 and 50 who presented with a unilateral torn ACL confirmed by functional testing who met the eligibility criteria were recruited. A total of twelve subjects were enrolled into the study. Patient characteristics are presented in Table 1.

**Table 1:** Patient characteristics

Patient characteristics	
Patients (N)	**12**
Age median (Range)	**29 (18–49)**
Male/female (N)	**6/6**
BMI	**24.3 (3.4)**
Smoker/non-smoker	**3/9**
Cause of ACL Injury	
- Contact sport (N)	**8/12**
Time from Injury to Surgery (months)	**44**
Meniscus injury (N)	**6/12**
Cartilage Injury ICRS> 2 (N)	**0/12**

### Inclusion and exclusion criteria

Inclusion Criteria:

- ACL tear requiring surgical reconstruction with semitendinosus and gracilis graft.
- Willing and able to give voluntary informed consent to participate in this study.
- Willing and able, in the opinion of the investigator, to cooperate with study procedures and willing to return to the study site for all post-operative study visits.
- Subject between 18 and 50 years at the time of surgery.
- ASA group 0–2 (limited medical illness).

Exclusion Criteria:

- Revision ACL reconstruction.
- Cartilage injury (IKDC Grade IV lesion > 2 cm2).
- Current malignant disease.
- Rheumatoid arthritis.
- Osteonecrosis or Avascular Necrosis or Ankylosing spondylitis.
- Subject obese (Body Mass Index [BMI] > 35).
- Subject pregnant or plans to become pregnant during the study.
- Subject had received medical treatment within 6 weeks of enrollment with any of the following: Glucocorticoids or Growth hormone.
- Participation in another investigational trial

### Surgery

All patients had ACL reconstruction using arthroscopically assisted technique. Gracilis and semitendinosus tendons were harvested through a small incision over the pes anserinus. All four strands were sutured separately with No 2 non resorbable sutures. The grafts were placed with an Endobutton CL implant (Smith&Nephew, Andover, MA, USA) for femoral fixation. The Endobutton loop length ensured that a minimum 25 mm of the graft was placed in the femoral tunnel.

**Fig. 1:**
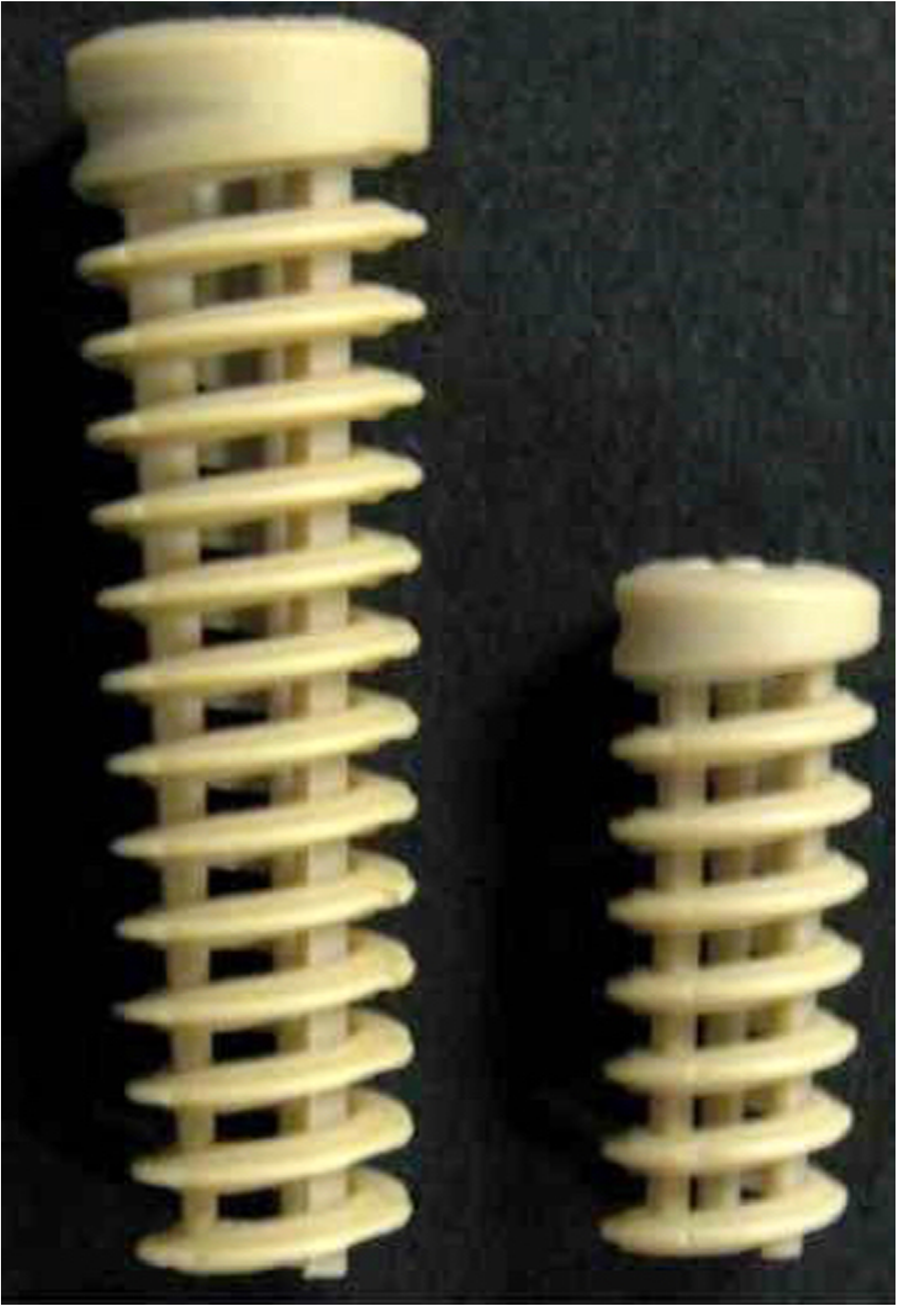
The BIOSURE HEALICOIL PK interference screw implant. The implant is characterized by three central columns and an open thread architecture allowing bone ingrowth into in between threads and screw center

Femoral drilling was performed through an anteromedial portal with the drillhole placed centrally in the femoral ACL footprint. A 55-degree oblique tibial drillhole into the anterior tibial cortex between the anterior edge of the medial collateral ligament and the tibial tuberosity was drilled ending in the center of the ACL tibial footprint. Drillhole diameter was sized according to the diameter of the four-strand graft with half millimeter incremenets The four-strand graft was pulled in place. In all patients, the tibial fixation was performed with the Biosure Healicoil interference screw (Smith&Nephew, Andover, USA) with a diameter of the tibial drillhole. The screw has an open thread design that allows bone ingrowth into the tread openings and screw center (Fig. 1). The screw is composed of polyetheretherketone (PEEK). The screw was placed just inside the tibial cortex for optimal graft compression and fixation in the tibial tunnel. Graft twisting or graft damage was visually inspected during screw positioning.

The incision was closed in layers and low suction drains were inserted into the joint and graft harvest area. The patients were discharged from the hospital 2–4 h postoperatively.

### Postoperative rehabilitation

The knee was allowed free range of motion from day one, followed by isometric quadriceps and passive flexion exercises. Patients were allowed full weight bearing as tolerated by pain and effusion using crutches for the first two post-operative weeks. Stationary bike exercises were used from the fourth postoperative week and progressive quadriceps strengthening exercises were conducted from the sixth week. Running was allowed at three months postoperatively, followed by return to lateral actions and contact sports at twelve months postoperatively or later. Rehabilitation was physiotherapist supervised for three months and used criterion-based activity progression.

### Outcome evaluation

CT scanning evaluated bone ingrowth into the Biosure Healicoil interference screw. Patient reported outcome and knee stability evaluation were performed after six, twelve and 24 months during a project control visit. An independent physiotherapist performed objective knee examinations.

#### Primary outcome CT scanning

The primary endpoint was visible bony ingrowth on six and twelve -month CT scans. CT scans to determine the presence of bone ingrowth into implant tread openings or central cavity were performed at two weeks, six and twelve months post-operatively. In order to be categorized as having bony ingrowth, a subject must have bone ingrowth of 4% or greater compared with baseline in the volume of the screw.

The patients were scanned in a supine position in a multislice CT-scanner (Brilliance 64-slice, Philips Medical Systems, Cleveland, OH). Axial slices were made starting from the joint-space level to one centimeter distal to the cap of the screw. The scans were performed in high resolution with collimating on 64 × 0.625 mm, slice thickness 0.9 mm. Reformatting was performed a with C-filter resulting in a slice thickness of 0.5 mm and 1.0 mm spacing.

#### CT scanning analysis

**Fig. 2:**
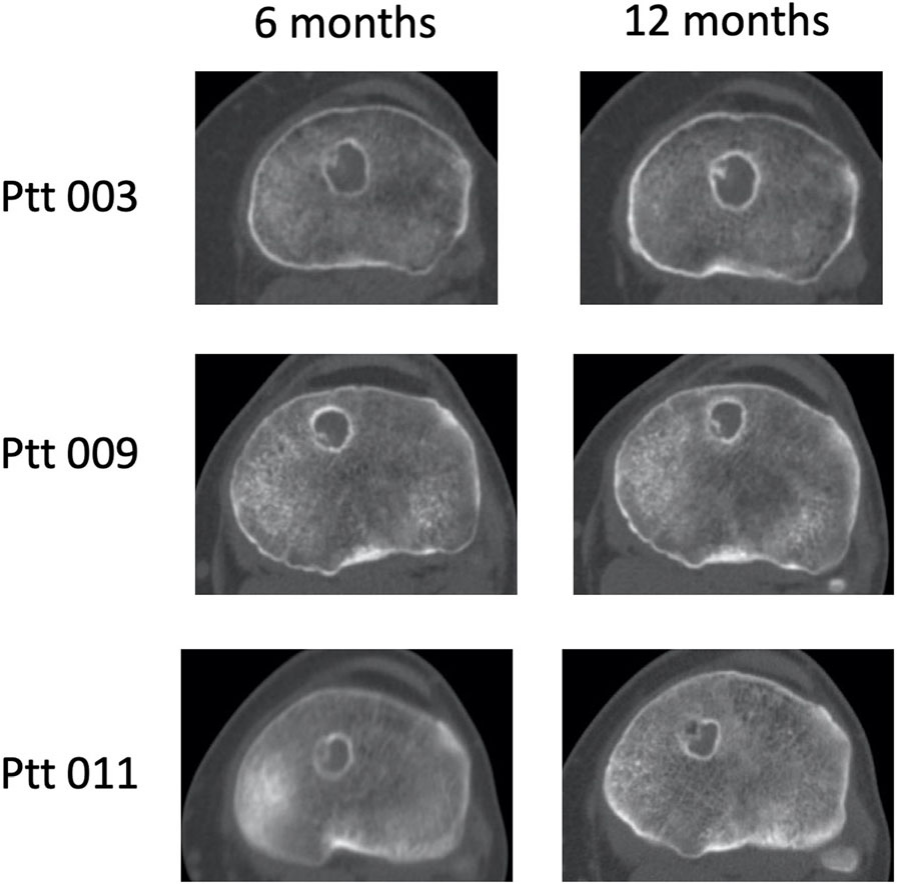
CT scanning images of characteristic bone ingrowth pattern. Three examples of average ingrowth at 6 and 12 months follow-up are shown

The same HU was used across all subjects/timepoints. Hounsfield Unit (HU) threshold for bone (threshold value was determined based on the average histogram value for trabecular bone across multiple subjects and timepoints) to generate a single value. For each subject timepoint, the highest spatial resolution DICOM series was converted to a single volume (VFF file format). All other timepoints for this particular subject were then volumetrically registered to this realigned Timepoint A using a mutual information algorithm. The resulting registered volumes were then re-sampled to conform with the voxel resolution of the realigned Time-point A using tri-linear interpolation. In this manner, all of the subject images were aligned with a standard orientation, and within each subject data set all of the timepoints had the same spatial resolution. This enabled direct comparison of all imaging timepoints for all subjects. For analysis of bone volume within the tunnel, a model of the Biosure Healicoil screw (size-specific for each subject) was fitted to each subject’s CT volume (mutual information based spatial registration). Voxels representing bone within the screw volume were subsequently segmented using a fixed Bone volume (sum of segmented bone voxels) and screw volume were calculated (Fig. 2). In order to be categorized as having bony ingrowth, a subject must have bone ingrowth of 4% or greater in the screw volume compared to baseline. This to account for any minor bone material entering the screw threads during insertion. A 10% bone ingrowth compared to baseline was arbitrary chosen as a good bone ingrowth and ingrowth data are presented as both average bone ingrowth and proportions of good ingrowth. Tunnel widening was assessed by measuring tunnel diameter on the immediate post-op CT scanning to follow-up CT scanning.

#### Secondary outcomes

Secondary endpoints were the following. Objective knee stability evaluated as maximal sagittal knee translation measured by KT-1000 arthrometer (MEDmetric, San Diego, CA) [2]. Pivot shift test with presentation of patients without a positive pivot shift test postoperatively. Subjective outcome scores as evaluated by subjective International Knee Documentation Committee (IKDC) score ranging from 0 to 100 with 100 being the score representing completely normal knee function [12]. Sports related knee function was evaluated by Tegner activity scale ranging from 0 to 10 with 10 being the score representing sports activity a professional soccer level [24].

### Safety and adverse events

Adverse and serious adverse events were registreted throughout the follow-up period.

Prior to enrollment, all subjects gave informed consent. The study was approved by the local scientific ethical committee (Region XXXX Ethical Committee Approval no. M-20110138) and by the XXXX Data Protection Agency and conducted in accordance with the Helsinki Declaration.

### Statistical analysis

#### Determination of sample size

CT scanning was used to document bone ingrowth at six and twelve months follow-up. A proportion of 50% of implants with bone ingrowth at twelve months was considered a clinically relevant outcome. Ten subjects were estimated to provide a 91% power for detecting a statistically significant difference (i.e. p < two-side alpha = 0.05) when a proportion of bone ingrowth of 5/10 was detected versus an assumed proportion of 10% (i.e. a proportion that was defined as clinically irrelevant) at twelve -month CT scans. To account for enrollment dropout, twelve subjects were planned to be enrolled thus providing a 20% oversampling with a likelihood of obtaining 10 subjects available for analysis.

#### Data presentation and comparisons

CT scannning data is presented as mean and standard deviations. For comparison of bone ingrowth two-tailed paired T-test was used. *P*-values less than 0.05 were considered significant. For data summaries, categorical and ordinal variables were summarized using frequency and percent. Continuous variables were summarized with the following summary statistics: number of observations, mean, median, standard deviation, minimum and maximum values. All analyses were performed using SAS versions 9.3 and 9.4.

## Results

The median patient age at the time of operation was 29 years (18–49 years). All patients were available for CT scannings at six, twelve and 24 months follow-up.

### CT scanning bone ingrowth outcome

**Fig. 3:**
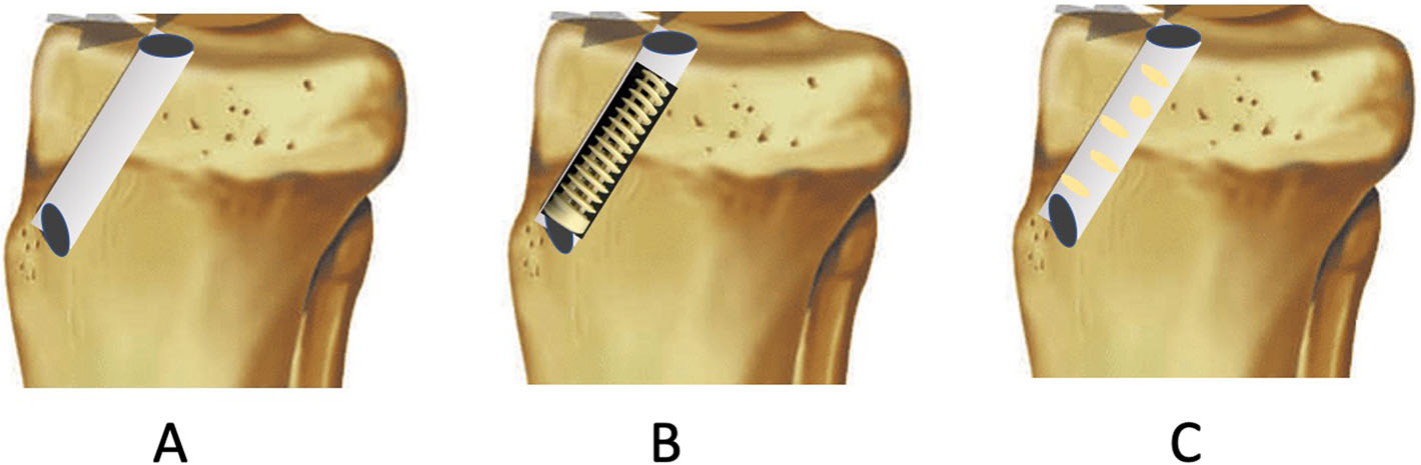
A graphically illustration of the three tibial volumens that are determined for CT evaluation of bone ingrowth into the Healicoil screws. **a**: Volumen of the tibial tunnel in the location of the Healicoil screw. **b**: Volume of the PEEK material of the Healicoil screw. **c**: Volumen of the new bone formation in the areas of screw voids. Bone ingrowth is determined as New bone volume C/Void volumen **a** – **b**

At 6 months no implant demonstrated more than 10% bone ingrowth. At twelve months 42% (5/12) implants had more than 10% bone ingrowth (*p* = 0.009) compared to no ingrowth. At 2 weeks (baseline) there was minor bone ingrowth average 0.9 (0.55) %. At six months, bone ingrowth averaged 3.1 (2.7)% and at twelve months bone ingrowth was 8.1% (4.5) (Fig. 2). The ingrowth average change from baseline to six months was 2.2 (2.8) % (*P* = 0.021) and at twelve months 7.7 (4.8)% (*P* = 0.002) Table 2. Representative CT images of bone ingrowth is presented in Fig. 3.

**Table 2:** Bone ingrowth data (% bone tissue inside screw) for all subjects at time zero, six and twelve months

Subjects	Baseline	6 months	12 months
1	1.5	0.1	1.1
2	1.0	2.7	4.8
3	0.0	6.4	10.9
4	0.5	3.8	12.2
5	1.5	0.4	0.2
6	0.5	1.8	7.5
7	1,7	0.6	7.4
8	0.1	0.4	7.3
9	1.4	8.8	12.9
10	0.8	5.0	12.1
11	0.8	3.7	14.0
12	0.9	3.7	7.2
Average	0.89 (0.6)	3.1 (2.7)	8.1 (4.5)

There was no tunnel widening or cyst formation observed in any patients.

### Clinical outcome

Subjective IKDC scores improved significantly from baseline to all follow-up time periods. The score at baseline was 50.6 and improved at 24 month follow-up to 80.1. The changes in the mean absolute values of the IKDC scores were statistically significant at both twelve months (23.1, *p* = 0.003) and 24 months (29.5, *p* < 0.001) (Table 3). Tegner Activity Scale scores also improved from a baseline mean level from median 2 to 4 at 24 months follow-up. At 24 months follow-up, 8 of 12 patients had a score of 4 or above. Most subjects (75%) had a baseline Tegner score of 2 (6 subjects) or 3 (3 subjects). Seven (7) of these subjects had improved by at least one point by six months; at twelve months all nine subjects’ improvement was one point or more and this was maintained through 24 months.

**Table 3:** Subjective IKDC score and Tegner Activty Scale scores at baseline and follow-up

**Subjective IKDC scores**				
	Baseline	6 Months	12 Months	24 Months
Mean (SD)	50.6 (19)	70.8 (16)^a^	73.7 (19)^a^	80.1 (18)^a^
**Tegner activity scale score**				
Median (Range)	2 (2–3)	4 (1–7)	4 (1–9)	5 (1–9)

^a^indicates significant difference from baseline to follow-up. (two-tailed Student T-test)

### Knee stability

Preoperative side to side knee laxity improved from 3.7 (2.1) 1.4 (1.2) mm (*p* = 0.004) at 12 months. A negative pivot shift was found in 11 patients and one patient had a slight positive pivot glide.

### Adverse events

There were eleven reported adverse events during the 24 month study period. These were: (1) postoperative pain, (3) pain in knee, (5) swelling/synovitis and (2) extension deficit. All events except one extension deficit condition resolved spontaneously with rest or supplementary oral analgesic medicine.

In one case, the extension deficit developed into a serious adverse event as the extension deficit did not resolve within 8 weeks post-operatively. Arthroscopic debridement of local anterior scar tissue formation was performed resulting in normalization of range of motion.

## Discussion

The primary finding of the present study was that a clinically relevant bone ingrowth into an open architecture PEEK interference screw in the tibial tunnel of subjects undergoing ACL reconstruction with soft tissue grafts occurred in 5 of the twelve subjects with an average bone ingrowth of 7.2% at twelve months postoperatively. There is no other litterature from clinical studies that have investigated bone ingrowth into open architecture non-absorbable interference screws after ACL reconstruction. One animal study investigated bone ingrowth and fixation strength after six months of perforated polylactategluconicacid (PLGA) screws in a sheep model and found bone ingrowth in the perforations and a tendency to increase fixation strength of the perforated screw compared to non-perforated screws [11]. In a clinical study aiming to investigate screw resorption of a poly-DL- Lactide (PDLLA) screws, perforations of the screw were used to accelerate the resorption of the screw but bone ingrowth was not investigated. The screw demonstrated complete resorption after 30 months [1]. Another study investigated the long-term screw remodelling and bone ingrowth in composite hydroxyapatite-polylactide (HA-PLLA) screws area. The study found that the HA-PLLA material stimulated osteoconductivity resulting in almost complete screw bone ingrowth after five years with 30% ingrowth after one year [13]. The higher bone formation was possible due to the resorbable nature of the screw which is different from the present fenestrated screw where the screw material remains intact. This probably the main reason the potential higher bone formation in an absorbable screw.

Secondary objectives of the present study were to assess changes in IKDC scores and Tegner Activity Scale scores from baseline to twelve and 24 months postoperatively. At both time points, there was statistically significant improvement from baseline. The IKDC score at 24 months was 80 which was similar to several large volume registry ACLR outcome studies [19, 22]. Similarly, functional outcome Tegner Activity scale score was 4.6 Another secondary outcome was knee stability which was found to improve significantly from a preoperative sagital laxity of 3.7 mm to a twelve months postoperative laxity of 1.4 mm.

There were no adverse events in relation to the usage of the open architechture interference screw during hamstring ACL reconstruction. Importantly no events of graft damage or twisting were observed during screw positioning. During the follow-up period a number of adverse events were observed that were typical for the intraarticular reaction to an ACL reconstruction and none of these adverse events were considered related to the tibial implant.

A strength of the study is that high resolution CT scanning was used to evaluate bone ingrowth. CT scanning is the golden standard for assessing bone healing and formation [7]. Several other studies have used MRI for evaluation of screw resorption and bone formation after the usage of resorbable interference screw and MR scanning might be sufficient to identify screw remnants but has a poor ability to identify newly formed calcified tissue [1, 18].

The clinical relevance of the present study is that open architecture interference screws used for tibia fixation in soft tissue ACL reconstruction induces bone ingrowth in almost half of cases. The bone healing activity may result in improved implant fixation and better graft incorporation into the bone tunnel although this clinical study did not investigate these factors. An open architecture implant inducing bone ingrowth could be beneficial in revision situations. In cases of traumatic rerupture of the ACL graft, a bone tunnel with an open architecture graft fixation implant consisting of PEEK and with bone ingrowth would not require implant removal during ACL revision. Drilling a new tunnel for the revision graft could then be performed throught the implant at whatever optimized tunnel position desired.

### Limitations

The present study has some limitations. With only twelve patients included, the design was sufficient to describe the desired biological phenomenon of bone ingrowth but lack the ability to generate complete data for clinical outcomes such as knee stability, subjective patient reported outcomes and failure rates but the clinical outcome data are primary included to identify adverse events. Another limitation is that no control group was included, but as no other fenestrated screw exist it is hard to have a relevant control group. As ingrowth screws is a novel concept been investigated, then there is no established standard value for clinically relevant bone ingrowth that can be used as threshold in study. We therefore had to choose a threshold for clinically relevant bone ingrowth, which we then sat at 10%.

## Conclusion

The present study demonstrated that open architecture thread PEEK interference screw can stimulate bone ingrowth into the screws after soft tissue ACL reconstruction with at twelve months with an average bone filling into screws was 7.7%. Knee stability, functional, subjective and objective outcomes were similar to large volume ACL outcome studies.

## Data Availability

Data are not available.
